# Example-Based Multispectral Photometric Stereo for Multi-Colored Surfaces

**DOI:** 10.3390/jimaging8040107

**Published:** 2022-04-11

**Authors:** Daisuke Miyazaki, Kazuya Uegomori

**Affiliations:** Graduate School of Information Sciences, Hiroshima City University, Hiroshima 731-3194, Japan; d20024@e.hiroshima-cu.ac.jp

**Keywords:** photometric stereo, color photometric stereo, example-based photometric stereo, multispectral imaging, multispectral lighting

## Abstract

A photometric stereo needs three images taken under three different light directions lit one by one, while a color photometric stereo needs only one image taken under three different lights lit at the same time with different light directions and different colors. As a result, a color photometric stereo can obtain the surface normal of a dynamically moving object from a single image. However, the conventional color photometric stereo cannot estimate a multicolored object due to the colored illumination. This paper uses an example-based photometric stereo to solve the problem of the color photometric stereo. The example-based photometric stereo searches the surface normal from the database of the images of known shapes. Color photometric stereos suffer from mathematical difficulty, and they add many assumptions and constraints; however, the example-based photometric stereo is free from such mathematical problems. The process of our method is pixelwise; thus, the estimated surface normal is not oversmoothed, unlike existing methods that use smoothness constraints. To demonstrate the effectiveness of this study, a measurement device that can realize the multispectral photometric stereo method with sixteen colors is employed instead of the classic color photometric stereo method with three colors.

## 1. Introduction

The photometric stereo method is not suitable for modeling a moving object since several images with different directions of the light source are needed. The color photometric stereo method can measure the shape of a moving object, which employs red, green, and blue lights in three different directions. Unlike the common color photometric stereo method, we use 16 narrow-band lights with different peak wavelengths while observing the target object with a 16-band multispectral camera.

### 1.1. Related Work

The shape-from-shading method [1,2,3,4,5,6] and the photometric stereo method [7,8] estimate the surface normal of an object by illuminating the object and analyzing the resulting shadings on the object’s surface. Unlike shape-from-shading, which uses one image, the photometric stereo captures three images with different light source directions. Therefore, it is impossible to measure a dynamic object. This problem can be resolved using the color photometric stereo method [9,10,11,12,13,14,15,16,17,18,19,20,21,22,23,24,25,26,27,28] (also known as shape-from-color). The color photometric stereo takes one picture with an RGB color camera under red, green, and blue light sources. Such a one-shot photograph enables the measurement of a dynamic object. However, the color photometric stereo has many problems. The major problem of the color photometric stereo method is the fact that it can only be used with white objects. This is an inevitable problem as long as lights are illuminated from colored light sources to estimate the surface normal.

Some methods [20,25,29] use multiple images to apply a color photometric stereo to multicolored objects. These methods cannot estimate the surface normal from a single image; thus, the optical flow method is used to track the identical point on the object surface among multiple images. Fyffe et al. [16] used three lights that can be recognized as white color by the human eye. The target objects are observed by a six-band camera. Each of the three lights has a different spectral distribution, which can be distinguished by the six-band camera. They estimate the surface normal without disturbing the human eye’s appearance. As conducted by Anderson et al. [9], using the shape from other methods such as multiview stereo enables the color photometric stereo to be applied to multicolored objects. Chakrabarti et al. [11] and Jiao et al. [19] assumed that a certain limited area has the same albedo. This assumption enables a color photometric stereo to be applied to multicolored objects that can be segmented for each colored region.

Example-based photometric stereos [30,31,32,33,34] estimate the surface normal using a database search. Those methods capture some images of objects with known shapes. They assume that the material properties of the objects in the database and the objects to be measured are the same. If the appearances of the pixels among those two types of objects are the same, these pixels might have the same surface normal. The example-based photometric stereo is used for a conventional photometric stereo problem, which assumes the same albedo for each light and is not used for the color photometric stereo problem since the albedo differs for each light.

### 1.2. Our Work

In this paper, the problem faced by the color photometric stereo method is solved using a different approach from those used in previous studies. We use the example-based photometric stereo to solve the problem of the color photometric stereo. Our approach solves the problem of shadow, specular reflection, and channel crosstalk.

Unlike Guo et al. [35], our method can be applied to the objects whose chromaticity and albedo are both spatially varying. The techniques of Gotardo et al. [29], Kim et al. [20], and Roubtsova et al. [25] need to employ optical flow to measure a dynamic object, while the technique of Fyffe et al. [16] requires a reflectance database to be prepared prior to the measurement. Our proposed technique does not require a shape obtained from other sensors such as a multi-view stereo or a laser sensor, unlike the technique of Anderson et al. [9]. Moreover, unlike the techniques of Chakrabarti et al. [11] and Jiao et al. [19], our proposed method does not require region segmentation. Our method is not oversmoothed by median filtering [36] and is not affected by randomness [37].

Previous color photometric stereo methods used three lights with red, green, and blue colors and observed the object with an RGB color camera. In our study, 16 lights with different wavelengths are used to illuminate the object, which is then observed by a 16-band multispectral camera. This paper empirically proves that the example-based photometric stereo is also useful for color photometric stereo situations.

Section 2 and Section 3 explain the fundamental theory of the color photometric stereo and example-based photometric stereo, respectively. Section 4 explains our example-based multispectral photometric stereo. Section 5 and Section 6 show the experimental results and the conclusion, respectively. In particular, Section 5.5 discusses the advantages and disadvantages of our method.

## 2. Color Photometric Stereo

A photometric stereo method that employs independent colored light is called the color photometric stereo method. A characteristic of this method is that it enables the estimation of the surface normal with one photoshoot. The widespread color photometric stereo method is conducted with three types of colored lights. While the conventional photometric stereo method results in several grayscale images, the color photometric stereo method results in a multi-spectral image.

Given n as a normal vector and $l_{c}$ as the light source direction vector of channel *c*, then the multispectral image can be:

(1) $$I_{c}=A_{c}\text{max}\left(n\cdot l_{c},0\right)\;.$$

Hereinafter, we call $A_{c}$ albedo. Note that the camera sensitivity and light source brightness are included in $A_{c}$.

**Figure 1:**
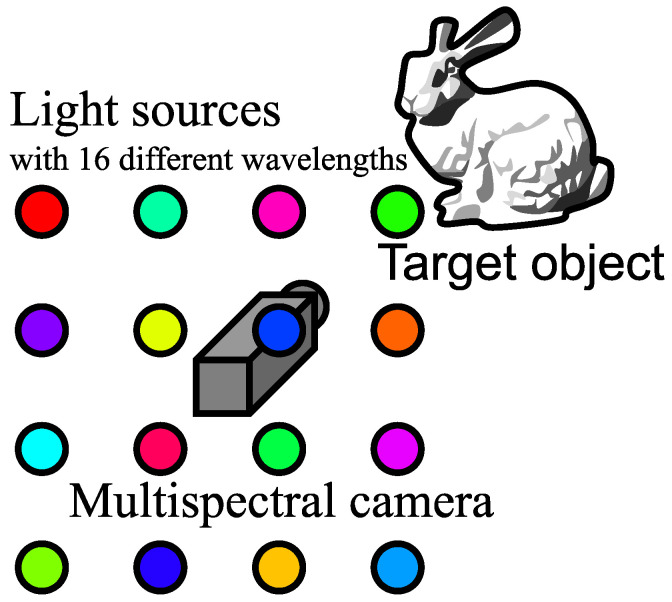
Conceptual explanation of multispectral color photometric stereo. Target object is illuminated by multiple light sources whose wavelengths are different. One image is taken using the multispectral camera.

As shown in Figure 1, this study conducts a photoshoot of a multicolored object using 16 channels. Following Equation (1), the brightness is obtained from this photoshoot as follows.

(2) $$\begin{array}{ccc} I_{0} & = & A_{0}\text{max}\left(n\cdot l_{0},0\right)\;,\\ I_{1} & = & A_{1}\text{max}\left(n\cdot l_{1},0\right)\;,\\ & \vdots & \\ I_{15} & = & A_{15}\text{max}\left(n\cdot l_{15},0\right)\;. \end{array}$$

The surface normal n is a 3D vector; however, the degree-of-freedom is two because it is constrained to be a unit vector (such constraint reduces one degree-of-freedom). Albedo $A_{c}$ is represented by 16 parameters. There are 16 equations, as shown in Equation (2), and 18 unknown parameters ($A_{0}$, $A_{1}$, ..., $A_{15}$, $n_{x}$, $n_{y}$, $n_{z}$, s.t., $n_{x}^{2}+n_{y}^{2}+n_{z}^{2}=1$, namely 16 for albedo and 2 for surface normal). Therefore, color photometric stereo is an ill-posed problem.

The most commonly used assumption is to limit the color of the target objects to white ($A_{0}=A_{1}=\cdots =A_{15}$). The color photometric stereo for white objects, or in other words, the conventional photometric stereo, can directly solve the surface normal without iterative optimization nor additional constraints, such as smoothness constraints. However, this paper analyzes the methods with multi-colored objects.

## 3. Example-Based Photometric Stereo

**Figure 2:**
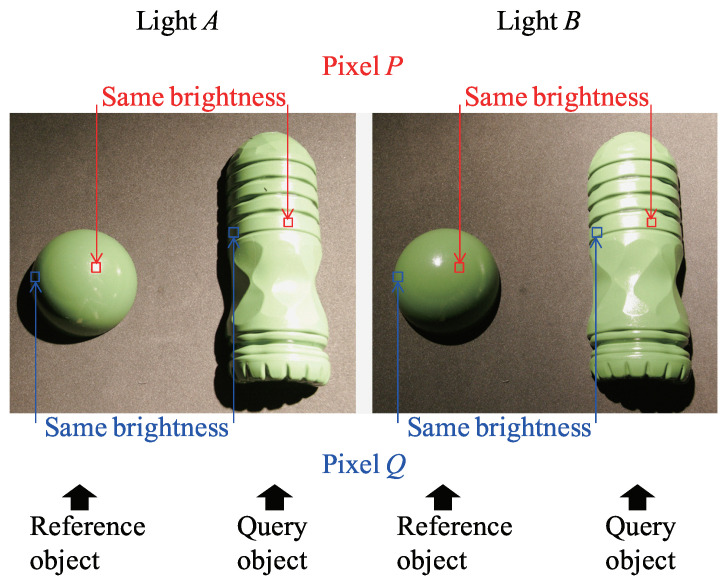
Brightness search of the example-based photometric stereo.

The example-based photometric stereo (Figure 2) uses the reference objects with known shapes for estimating the surface normal, which can be applied to non-Lambertian surfaces. The example-based photometric stereo measures two objects with known and unknown shapes under the same scene. Those two objects should have the same material property.

A sphere is often used for reference objects. Both brightnesses coincide if the surface normal of the target object and the surface normal of the reference object coincide because the material property, light direction, and camera direction are the same. Therefore, the example-based photometric stereo can estimate the surface normal of the objects with an arbitrary BRDF (bidirectional reflection distribution function). The disadvantage of the example-based photometric stereo is that the reference objects whose material property is the same as the target objects are needed. The interreflection between each surface point is not considered in this method.

## 4. Proposed Method

### 4.1. Example-Based Multispectral Photometric Stereo

Existing methods add some constraints such as smoothness to solve since the unknowns exceed the inputs. Such an approach oversmoothes the albedo and the surface normal. Our method does not require any constraints.

**Figure 3:**
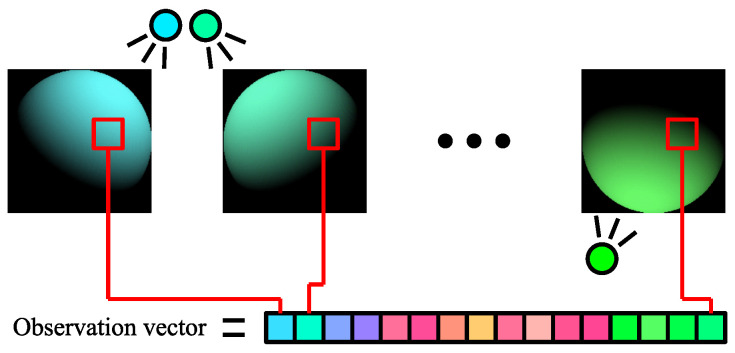
Observation vector.

We observe the object illuminated under 16 lights with different wavelengths using the multispectral camera (Figure 1). The observation vector at pixel $\left(y_{Q},x_{Q}\right)$ of query image (the image of target object) is denoted as ${\left(I_{Q,0},I_{Q,1},\cdots ,I_{Q,15}\right)}^{⊤}$ and the observation vector at pixel $\left(y_{R},x_{R}\right)$ of reference image (the image of the database) is denoted as ${\left(I_{R,0},I_{R,1},\cdots ,I_{R,15}\right)}^{⊤}$. If the query’s albedo ${\left(A_{Q,0},A_{Q,1},\cdots ,A_{Q,15}\right)}^{⊤}$ and the reference’s albedo ${\left(A_{R,0},A_{R,1},\cdots ,A_{R,15}\right)}^{⊤}$ coincide and the query’s observation vector and the reference’s observation vector coincide, the surface normal at $\left(y_{R},x_{R}\right)$ and the surface normal at $\left(y_{Q},x_{Q}\right)$ coincide. Each element of the 16-dimensional observation vector (Figure 3) is Equation (2).

**Figure 4:**
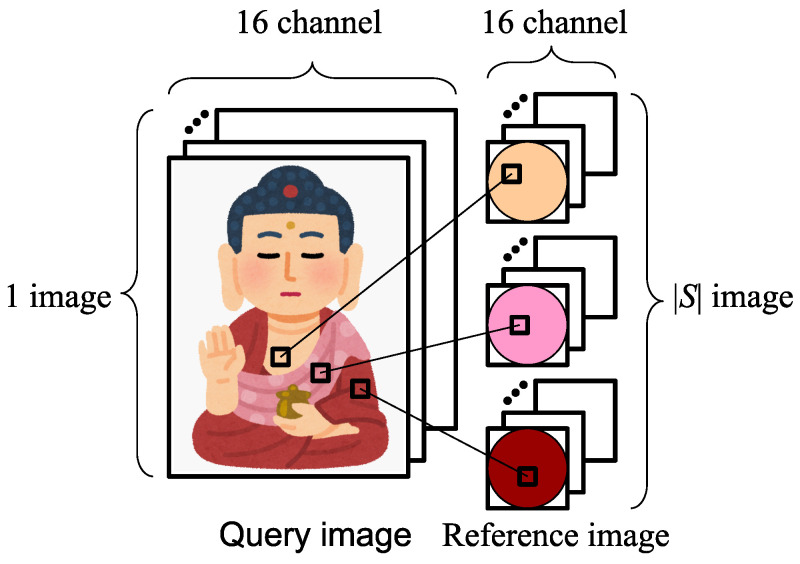
Our approach.

We search the pixel position of the reference object where the query’s observation vector coincides with the reference’s observation vector (Figure 4). The query’s surface normal is determined from the pixel position of the reference found. Multiple spheres with different paints are used as the reference. The search of the observation vector is performed for all pixels of all reference spheres.

Our method (Equation (3)) searches the pixel position where the squared error of the 16-dimensional vector becomes the minimum.

(3) $$\begin{array}{ccc} & & n\left(y_{Q},x_{Q}\right)=n_{R}\left(y_{R},x_{R}\right)\;,\\ & & s.t.\;\left(y_{R},x_{R}\right)=\underset{y_{R},x_{R}}{\mathrm{argmin}}\sum_{c\in C}{\left(I_{Q}\left(y_{Q},x_{Q},c\right)-I_{R}\left(s,y_{R},x_{R},c\right)\right)}^{2}\;,\;\forall s\in S\;,\;\forall \left(y_{R},x_{R}\right)\in P_{R}\;. \end{array}$$

Here, |C| is the number of channels (|C|=16), |S| is the number of reference objects, and $P_{R}$ is a set of reference’s pixels. We normalize the observation vectors of both the query image and the reference image. Thanks to the normalization, our method can be applied even if the camera exposure is changed.

In order to apply our method to any objects with any paints, we have to measure all paints in the world. However, the variation of paints is limited due to the limitation of chemical reactions. The number of paints is limited if the paints are based on pure natural pigments since the number of natural pigments is limited. In this paper, we assume that all paints can be expressed in a limited number. We used 18 spheres with different colors (|S|=18).

### 4.2. Converting Surface Normal to Height

The shape is represented as the height *H* set for each pixel. The partial derivatives of the heights with respect to *x* and *y* are called gradient and represented as *p* and *q*, respectively.

(4) $$p=H_{x}=\frac{\partial H}{\partial x}\;,\;q=H_{y}=\frac{\partial H}{\partial y}\;.$$

The surface normal n is represented by these gradients, as shown below.

(5) $$n=\frac{{\left(-p,-q,1\right)}^{⊤}}{\sqrt{p^{2}+q^{2}+1}}\;.$$

The cost function that relates the surface normal to the height is shown below.

(6) $$\int \;\;\int {\left(H_{x}-p\right)}^{2}+{\left(H_{y}-q\right)}^{2}dxdy\;.$$

We solve Equation (6) to calculate the height from the surface normal using existing techniques.

### 4.3. Channel Crosstalk

**Figure 5:**
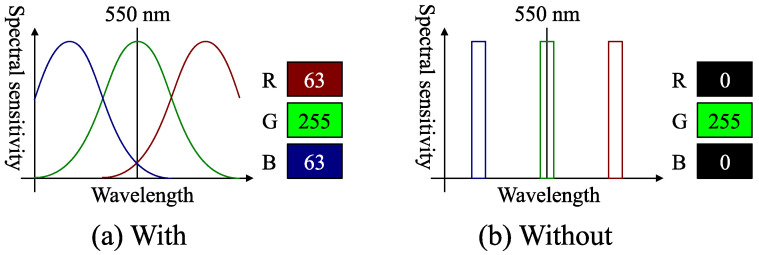
Example of camera spectral sensitivity: (**a**) the sensor that has channel crosstalk; (**b**) the sensor that does not have channel crosstalk.

The conventional color photometric stereo assumes that the camera spectral response is a delta function. Figure 5b is an example where only the G channel detects the 550 (nm) light. On the other hand, Figure 5a is an example where the sensor has channel crosstalks. Namely, the spectral responses of R, G, and B channels partially overlap in the spectral domain. In this example, the sensor detects (R,G,B)=(63,255,63) instead of (R,G,B)=(0,255,0) (Figure 5b) when 550 (nm) light is observed. Namely, the red and blue channels are excited even if the observed light is completely green. Such channel crosstalk is annoying for the conventional color photometric stereo. The conventional color photometric stereo assumes that, for example, only the green channel should detect the green light. Channel crosstalk commonly occurs in most cameras, which makes the color photometric stereo difficult. However, as discussed in Section 5.5, our method is free from the channel crosstalk problem.

## 5. Experiment

### 5.1. Experimental Setup

**Figure 6:**
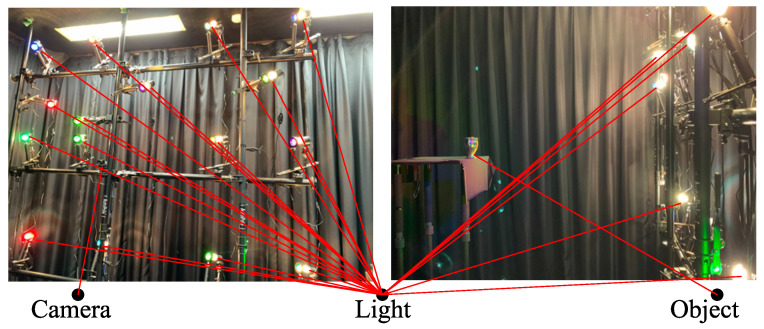
Experimental apparatus.

**Figure 7:**
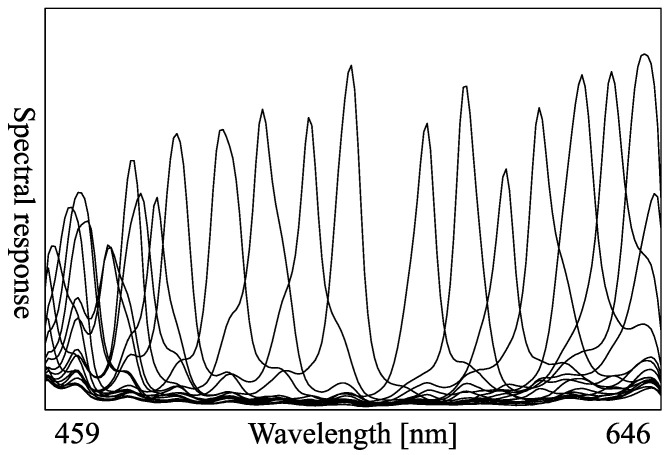
Spectral response of the camera.

**Figure 8:**
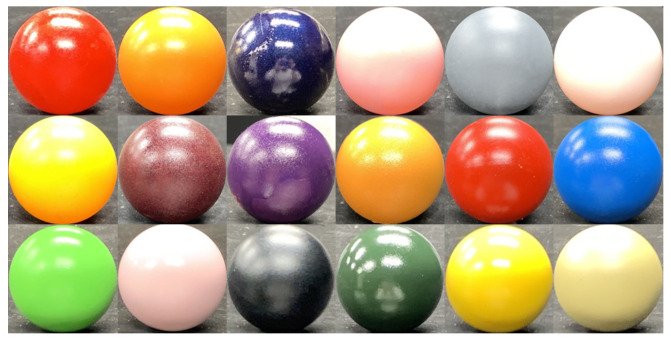
Reference objects.

We perform our experiment in a dark room, as shown in Figure 6, where the target object is illuminated under 16 different lights. We use IMEC-HS-16-USB-customized (Imec, Belgium) for the multispectral camera. Figure 7 and Table 1 show the spectral sensitivity of the camera, where channel crosstalks are occurring among all camera channels. Table 2 shows the peak wavelength for each light source used in this experiment. To increase the amount of supplementary information obtained for objects with narrow-wavelength regions, light sources of close wavelengths were positioned opposite to each other. Namely, as shown in Table 2, the light of the next larger wavelength is set far apart in more than one Manhattan distance in 4×4 grid. The locations of the light sources and the camera were left unchanged during the experiments. We assume that the light source and the camera are infinitely far from the target object. This paper represents the surface normal as pseudo-color, where *x*, *y*, and *z* of the normal vector are mapped to R, G, and B of the image. Each sphere image is trimmed and scaled to 128×128 size. The sphere objects shown in Figure 8 are painted with 18 different paints. The size of the query image is 512×256. The target object is opaque objects. Our method can estimate the surface normal of metals if the number of lights is infinity, but it cannot estimate with a finite number of lights. Transparent objects are more difficult to measure due to the transmission.

### 5.2. Evaluation

**Figure 9:**
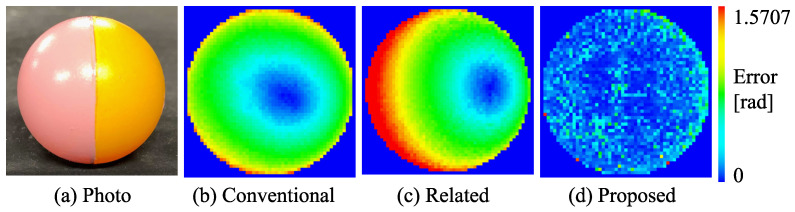
Performance evaluation result: (**a**) target spherical object with 2 paints; (**b**) the error map of the conventional photometric stereo; (**c**) the error map of the existing method; (**d**) the error map of the proposed method.

First, we measured a spherical object, shown in Figure 9a, consisting of two types of albedos painted with the paints included in the reference objects. The error is evaluated as an angle between the estimated surface normal and the true surface normal. We have to compare the estimated surface normal with the true surface normal by measuring the object whose true surface normal is known. We measured a sphere for evaluation. The mathematically true surface normal can be theoretically derived from the sphere’s center and radius. Suppose that the pixel of interest is (x,y) and the center of the sphere is $\left(\bar{x},\bar{y}\right)$. Suppose that the radius of the sphere is *r*. Then, the true surface normal $\left(n_{x},n_{y},n_{z}\right)$ can be calculated as follows:

(7) $$\begin{array}{ccc} n_{x} & = & \left(x-\bar{x}\right)/r\;, \end{array}$$

(8) $$\begin{array}{ccc} n_{y} & = & -\left(y-\bar{y}\right)/r\;, \end{array}$$

(9) $$\begin{array}{ccc} n_{z} & = & \sqrt{1-n_{x}^{2}-n_{y}^{2}}\;. \end{array}$$

Since we know the true surface normal from Equations (7)–(), we can evaluate the performance of the method by measuring a sphere. Figure 9b–d show the error map with pseudo-color representation. We compared our method with the conventional photometric stereo (Figure 9b). The color photometric stereo that assumes white objects as targets is the same as the conventional photometric stereo. Furthermore, we compared our method with an existing method [35] (Figure 9c). The error of the conventional photometric stereo (color photometric stereo with white object) was 0.690 (rad), the error of existing method (Guo et al. [35]) was 0.888 (rad), and the error of our method was 0.198 (rad), which proves the high performance of our method.

### 5.3. Real Objects

**Figure 10:**
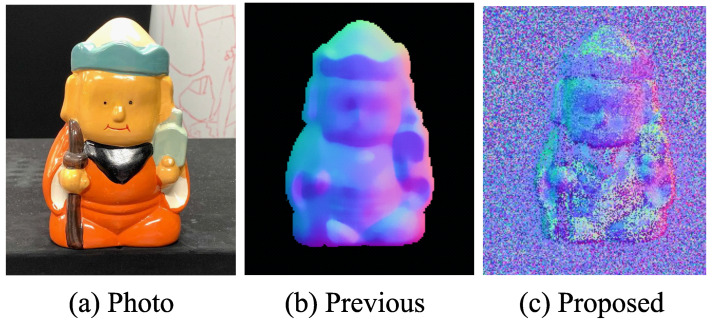
Comparison: (**a**) target object, (**b**) the estimated surface normal of previous method, and (**c**) the estimated surface normal of proposed method.

We apply the existing method [36] and our method to the object shown in Figure 10a. The estimated surface normals of the existing and proposed methods are shown in Figure 10b,c, respectively. Here, the surface normal of *x*, *y*, and *z* axes are represented as red, green, and blue colors. Unlike the existing method, which oversmoothes the result (Figure 10b), our method is a pixelwise approach, and the result is not oversmoothed (Figure 10c). The existing method [36] needs to segment the object region from the background (Figure 10b), while our method does not need to distinguish the foreground and the background. The existing method cannot estimate the surface normal of the background, while our method can; however, the surface normal of the background is just noise since the background has no object with a completely dark void and random noise (Figure 10c).

**Figure 11:**
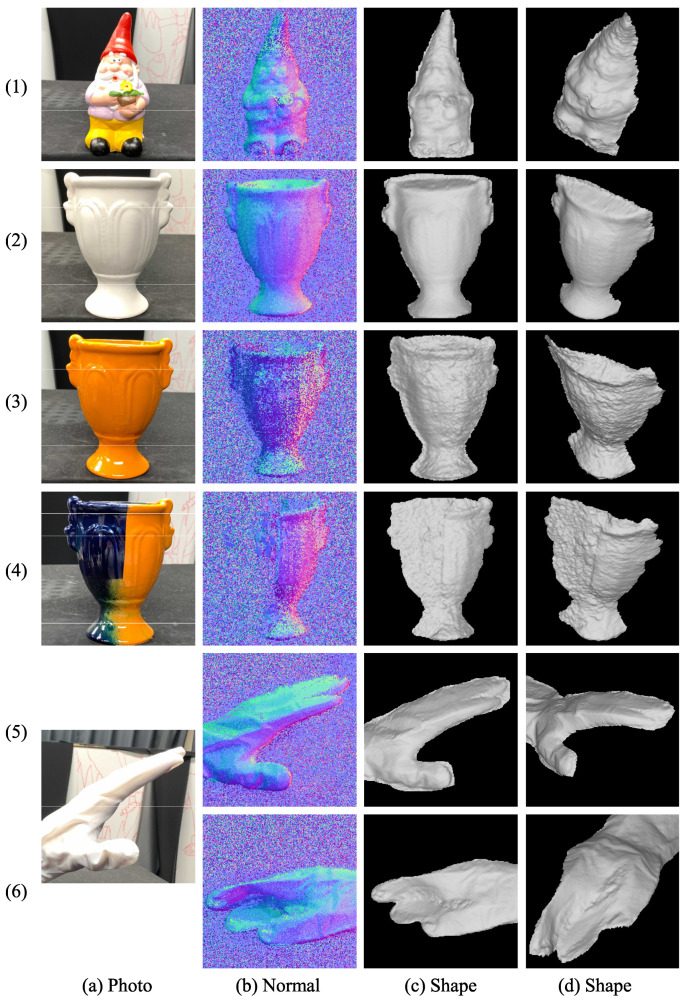
Experimental results of the (1) multi-colored object, (2) white object, (3) single-colored object, (4) dark object, and (5,6) deformable object: (**a**) Target object, (**b**) estimated surface normal, and (**c**,**d**) reconstructed shape.

The target objects are shown in Figure 11a. The paints used in Figure 11(3,4) are included in the reference data, while the others are not. The results of a multi-colored object, a white object, a single-colored object, an object with dark color, and a deformable object with two different poses are shown in Figure 11(1)–(6), respectively. The estimated surface normals of our method are shown in Figure 11b. Figure 11c,d show the reconstructed shapes under two different viewing directions. The quantitative evaluation shown in Section 5.2 proves the benefit of our method, and the qualitative evaluation shown in Figure 11 also proves the benefit of our method. As shown in Figure 11, our method can successfully estimate the surface normals for both achromatic (Figure 11(2)) and chromatic (Figure 11(1)) objects without oversmoothing them.

### 5.4. Discussion

**Figure 12:**
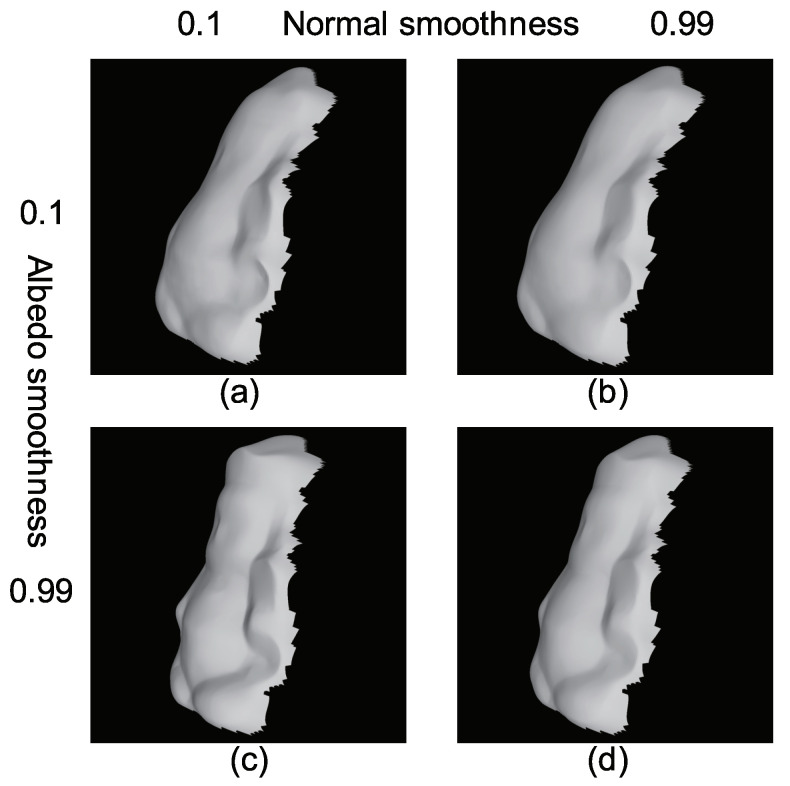
Parameter tuning problem of previous method: (**a**) sharp normal and sharp albedo; (**b**) smooth normal and sharp albedo; (**c**) sharp normal and smooth albedo; (**d**) smooth normal and smooth albedo.

We did not to add smoothness constraints, and thus, our result is not oversmoothed. Adding smoothness constraints results in smoother results, which are often required by the users. If we add some constraints, we have to tune the parameters of those constraints. Figure 12 shows the parameter tuning problem that occurred in the existing method [36]. In our future work, we would like to add smoothness constraints, but we have to carefully design the algorithm because adding smoothness constraints is not always a good approach due to the oversmoothing and parameter tuning.

Our method is applicable to multi-colored objects, as shown in the experiments, where error did not occur at the color boundary of the object (Figure 11(1)). Our method is robust to specular reflection, as shown in the experiments, where a spike-like error did not appear in the result (Figure 9c). Our method cannot estimate the surface normal of the dark surface; however, this disadvantage is always true to all other photometric stereo methods (Figure 11(4)).

### 5.5. Contribution

Here, we summarize our advantages and disadvantages.

Our method does not suffer from channel crosstalk since the reference object includes the information of channel crosstalk, and the query object and the reference object are measured under the same light and the same camera. Namely, our method is not affected by the spectral distribution of the lights and the spectral/radiometric response of the camera since both the query object and reference object are measured under the same lights and with the same camera. Our process is pixelwise, and thus, the result is not affected by neighboring pixels. The light source direction does not need to be measured because the target and reference objects are illuminated under the same illumination environment. Furthermore, we do not adjust each light source to be the same intensity. Our method is not limited to a Lambertian surface, and our method is not affected by shadows. If we prepare reference objects with specular reflection, our method can be applied to the objects with specular reflection.

The disadvantage of our method is that we need many reference objects. Furthermore, we have to measure the query object with the same device that the reference objects are taken since the light and the camera information are included in the reference objects.

The number of reference objects is related to both advantages and disadvantages. If we increase reference objects, our method can be applied to various types of paints. However, a similar observation vector might appear in the database if we increase reference objects. These are the characteristics of the example-based multispectral photometric stereo compared to the example-based conventional photometric stereo. The albedo $A_{0},A_{1},\cdots ,A_{15}$ has 16 degrees-of-freedom in our method but has 1 degree-of-freedom in the example-based photometric stereo. Due to the wider degrees-of-freedom, the unique database search is disabled if we use many reference objects. This is the dilemma of our method whether we should increase or decrease the number of reference objects.

## 6. Conclusions

Our method estimated the surface normal of multi-colored objects using 16 lights. The light source directions of all lights do not need to be measured. The query and reference objects are observed by a multispectral camera. We measured many spheres painted with a single color with various paints. Surface normals are the same for the two points on the surface if the material properties are the same, the light source directions are the same, and the camera direction is the same. We estimated the surface normal of the target object by finding the pixel where the data of the query image become the same as the data of the reference images.

Our experimental results show that our method has successfully estimated the surface normal of multi-colored objects. However, the dark albedo has caused some errors.

This time, we scanned all reference objects. However, it is well known that the spectral reflectance of any paint can be represented by a small number of basis functions. We conjecture that the bases of the PCA (principal component analysis) can represent the data with a small number of basis functions. Our future work is to install PCA in our method. 

## Figures and Tables

**Table 1:** The spectral response for each channel of the camera.

Channel 1	Channel 2	Channel 3	Channel 4
Peak 488 nm	Peak 499 nm	Peak 479 nm	Peak 469 nm
Peak 50% 488–492 nm	Peak 50% 495–503 nm	Peak 50% 467–486 nm	Peak 50% 464–474 nm
Channel 5	Channel 6	Channel 7	Channel 8
Peak 599 nm	Peak 609 nm	Peak 587 nm	Peak 575 nm
Peak 50% 459–465, 595–602 nm	Peak 50% 464–470, 606–615 nm	Peak 50% 583–591 nm	Peak 50% 570–578 nm
Channel 9	Channel 10	Channel 11	Channel 12
Peak 641 nm	Peak 644 nm	Peak 631 nm	Peak 622 nm
Peak 50% 483–488, 635–646 nm	Peak 50% 489–497, 637–646 nm	Peak 50% 626–638 nm	Peak 50% 468–473, 616–627 nm
Channel 13	Channel 14	Channel 15	Channel 16
Peak 539 nm	Peak 552 nm	Peak 525 nm	Peak 513 nm
Peak 50% 535–543 nm	Peak 50% 547–555 nm	Peak 50% 521–532 nm	Peak 50% 509–519 nm

**Table 2:** Peak wavelength of each light (10 nm width).

Light 1	Light 2	Light 3	Light 4
488 nm	632 nm	540 nm	500 nm
Light 5	Light 6	Light 7	Light 8
647 nm	600 nm	470 nm	610 nm
Light 9	Light 10	Light 11	Light 12
520 nm	568 nm	620 nm	473 nm
Light 13	Light 14	Light 15	Light 16
636 nm	515 nm	589 nm	550 nm
